# Directed Reachability for Infinite-State Systems

**DOI:** 10.1007/978-3-030-72013-1_1

**Published:** 2021-02-26

**Authors:** Michael Blondin, Christoph Haase, Philip Offtermatt

**Affiliations:** 8 Eindhoven University of Technology, Eindhoven, The Netherlands; 9 Aalborg University, Aalborg East, Denmark; 10 Université de Sherbrooke, Sherbrooke, Canada; 11 University of Oxford, Oxford, United Kingdom; 12 Max Planck Institute for Software Systems, Saarbrücken, Germany

**Keywords:** Petri nets, reachability, shortest paths, model checking

## Abstract

Numerous tasks in program analysis and synthesis reduce to deciding reachability in possibly infinite graphs such as those induced by Petri nets. However, the Petri net reachability problem has recently been shown to require non-elementary time, which raises questions about the practical applicability of Petri nets as target models. In this paper, we introduce a novel approach for efficiently semi-deciding the reachability problem for Petri nets in practice. Our key insight is that computationally lightweight over-approximations of Petri nets can be used as distance oracles in classical graph exploration algorithms such as $\mathsf {A}^{*}$ and greedy best-first search. We provide and evaluate a prototype implementation of our approach that outperforms existing state-of-the-art tools, sometimes by orders of magnitude, and which is also competitive with domain-specific tools on benchmarks coming from program synthesis and concurrent program analysis.

## 1 Introduction

Many problems in program analysis, synthesis and verification reduce to deciding reachability of a vertex or a set of vertices in infinite graphs, *e.g.*, when reasoning about concurrent programs with an unbounded number of threads, or when arbitrarily many components can be used in a synthesis task. For automated reasoning tasks, those infinite graphs are finitely represented by some mathematical model. Finding the right such model requires a trade-off between the two conflicting goals of maximal expressive power and computational feasibility of the relevant decision problems. Petri nets are a ubiquitous mathematical model that provides a good compromise between those two goals. They are

* An extended version containing full proofs as well as a primer on applications of the Petri net reachability problem can be obtained from: arxiv.org/abs/2010.07912. This work is part of a project that has received funding from the European Research Council (ERC) under the European Union's Horizon 2020 research and innovation programme (Grant agreement No. 852769, ARiAT). It is also supported by a Discovery Grant from the Natural Sciences and Engineering Research Council of Canada (NSERC). Parts of this research were carried out while the second author was affiliated with the Department of Computer Science, University College London, UK.

expressive enough to find a plethora of applications in computer science, in particular in the analysis of concurrent processes, yet the reachability problem for Petri nets is decidable [47,40,41,43]. *Counter abstraction* has evolved as a generic abstraction paradigm that reduces a variety of program analysis tasks to problems in Petri nets or variants thereof such as well-structured transition systems, see *e.g.* [30,39,61,5]. Due to their generality and versatility, Petri nets and their extensions find numerous applications also in other areas, including the design and analysis of protocols [22], business processes [57], biological systems [33,11] and chemical systems [2]. The goal of this paper is to introduce and evaluate an efficient generic approach to deciding the Petri net reachability problem on instances arising from applications in program verification and synthesis.

A Petri net comprises a finite set of *places* with a finite number of *transitions*. Places carry a finite yet unbounded number of *tokens* and transitions can remove and add tokens to places. A *marking* specifies how many tokens each place carries. An example of a Petri net is given on the left-hand side of Figure 1, where the two places $\{p_1, p_2\}$ are depicted as circles and transitions $\{t_1, t_2, t_3\}$ as squares. Places carry tokens depicted as filled circles; thus $p_1$ carries one token and $p_2$ carries none. We write this as $[p_1\colon 1, p_2\colon 0]$, or $(1, 0)$ if there is a clear ordering on the places. Transition $t_1$ can add a single token to place $p_1$ at any moment. As soon as a token is present in $p_1$, it can be consumed by transition $t_2$, which then adds a token to place $p_2$ and puts back one token to place $p_1$. Finally, transition $t_3$ consumes tokens from $p_1$ without adding any token at all.

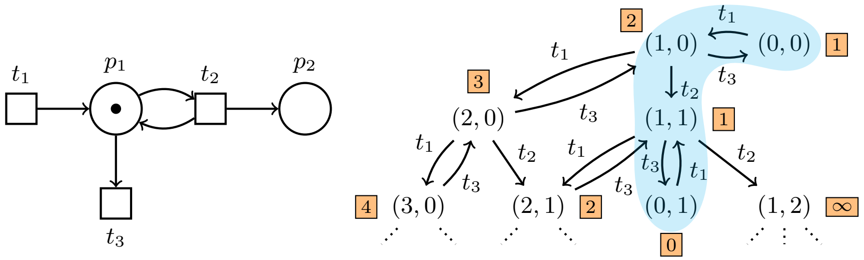

**Fig. 1.** *Left:* A Petri net $\mathcal{N}$. *Right:* Search of the forthcoming Algorithm 1 over the graph $G_{\mathbb{N}}(\mathcal{N})$ from $(0, 0)$ to $(0, 1)$, where $(x, y)$ denotes $[p_1\colon x, p_2\colon y]$ and each number in a box next to a marking is its heuristic value. Only the blue region is expanded.

A Petri net induces a possibly infinite directed graph whose vertices are markings, and whose edges are determined by the transitions of the Petri net, *cf.* the right side of Figure 1. Given two markings, the *reachability problem* asks whether they are connected in this graph. In Figure 1, the marking $(0, 1)$ is reachable from $(0, 0)$, *e.g.*, via paths of lengths 3 and 5: $(0, 0) \xrightarrow{t_1} (1, 0) \xrightarrow{t_2} (1, 1) \xrightarrow{t_3} (0, 1)$ and $(0, 0) \xrightarrow{t_1} (1, 0) \xrightarrow{t_1} (2, 0) \xrightarrow{t_2} (2, 1) \xrightarrow{t_3} (1, 1) \xrightarrow{t_3} (0, 1)$.

In practice, the Petri net reachability problem is a challenging decision problem due to its horrendous worst-case complexity: an exponential-space lower bound was established in the 1970s [45], and a non-elementary time lower bound has only recently been established [13]. One may thus question whether a problem with such high worst-case complexity is of any practical relevance, and whether reducing program analysis tasks to Petri net reachability is anything else than merely an intellectual exercise. We debunk those concerns and present a technique which decides most reachability instances appearing in the wild. When evaluated on large-scale instances involving Petri nets with thousands of places and tens of thousands of transitions, our prototype implementation is most of the time faster, even up to several orders of magnitude on large-scale instances, and solves more instances than existing state-of-the-art tools. Our implementation is also competitive with specialized domain-specific tools. One of the biggest advantages of our approach is that it is extremely simple to describe and implement, and it readily generalizes to many extensions of Petri nets. In fact, it was surprising to us that our approach has not yet been discovered. We now describe the main observations and techniques underlying our approach.

Ever since the early days of research in Petri nets, state-space over-approximations have been studied to attenuate the high computational complexity of their decision problems. One such over-approximation is, informally speaking, to allow places to carry a negative number of tokens. Deciding reachability then reduces to solving the so-called *state equation*, a system of linear equations associated to a Petri net. Another over-approximation are *continuous Petri nets*, a variant where places carry fractional tokens and "fractions of transitions" can be applied [14]. The benefit is that deciding reachability drops down to polynomial time [25]. While those approximations have been applied for pruning search spaces, see *e.g.* [23,4,8,29], we make the following simple key observation:

> *If a marking* $\boldsymbol{m}$ *is reachable from an initial marking in an over-approximation, then the length of a shortest witnessing path in the over-approximation lower bounds the length of a shortest path reaching* $\boldsymbol{m}$*.*

The availability of an oracle providing lower bounds on the length of shortest paths between markings enables us to appeal to classical graph traversal algorithms which have been highly successful in artificial intelligence and require such oracles, namely $\mathsf{A}^*$ and greedy best-first search, see *e.g.* [52]. In particular, determining the length of shortest paths in the over-approximations described above can be phrased as optimization problems in (integer) linear programming and optimization modulo theories, for which efficient off-the-shelf solvers are available [32,7]. Thus, oracle calls can be made at comparably modest computational cost, which is crucial for the applicability of those algorithms. As a result, a large class of existing state-space over-approximations can be applied to obtain a highly efficient forward-analysis semi-decision procedure for the reachability problem. For example, in Figure 1, using the state equation as distance oracle, $\mathsf{A}^*$ only explores the four vertices in the blue region and directly reaches the target vertex, whereas a breadth-first search may need to explore all vertices of the figure and a depth-first search may even not terminate.

In theory, our approach could be turned into a decision procedure by applying bounds on the length of shortest paths in Petri nets [44]. However, such lengths can grow non-elementarily in the number of places [13], and just computing the cut-off length will already be infeasible for any Petri net of practical relevance. It is worth mentioning that, in practice, it has been observed that the over-approximations we employ also often witness non-reachability though, see *e.g.* [23]. Still, when dealing with finite state spaces, our procedure is complete.

A noteworthy benefit of our approach is that it enables finding *shortest* paths when $\mathsf{A}^*$ is used as the underlying algorithm. In program analysis, paths usually correspond to traces reaching an erroneous configuration. In this setting, shorter error traces are preferred as they help understanding why a certain error occurs. Furthermore, in program synthesis, paths correspond to synthesis plans. Again, shorter paths are preferred as they yield shorter synthesized programs. In fact, we develop our algorithmic framework for *weighted* Petri nets in which transitions are weighted with positive integers. Classical Petri nets correspond to the special instance where all weights are equal to one. Weighted Petri nets are useful to reflect cost or preferences in synthesis tasks. For example, there are program synthesis approaches where software projects are mined to determine how often API methods are called to guide a procedure by preferring more frequent methods [27,26,46]. Similarity metrics can also be used to obtain costs estimating the relevance of invoking methods [24]. It has further been argued that weighted Petri nets are a good model for synthesis tasks of chemical reactions as they can reflect costs of various chemical compounds [58]. Finally, weights can be viewed as representing an amount of time it takes to fire a transition, see *e.g.* [50].

*Related work.* Our approach falls under the umbrella term *directed model checking* coined in the early 2000s, which refers to a set of techniques to tackle the state-explosion problem via guided state-space exploration. It primarily targets disproving safety properties by quickly finding a path to an error state without the need to explicitly construct the whole state space. As such, directed model checking is useful for bug-finding since, in the words of Yang and Dill [60], *in practice, model checkers are most useful when they find bugs, not when they prove a property.* The survey paper [20] gives an overview over various directed model checking techniques for finite-state systems.

For Petri nets, directed reachability algorithms based on over-approximations as developed in this work have not been described. In [56], it is argued that exploration heuristics, like $\mathsf{A}^*$, can be useful for Petri nets, but they do not consider over-approximations for the underlying heuristic functions. The authors of [36] use Petri nets for scheduling problems and employ the state equation, viewed as a system of linear equations over $\mathbb{Q}$, in order to explore and prune reachability graphs. This approach is, however, not guaranteed to discover shortest paths. There has been further work on using $\mathsf{A}^*$ for exploring the reachability graph of Petri nets for scheduling problems, see, *e.g.*, [42,48] and the references therein.

## 2 Preliminaries

Let $\mathbb{N} \coloneqq \{0, 1, \ldots\}$. For all $\mathbb{D} \subseteq \mathbb{Q}$ and $\succ \in \{\geq, >\}$, let $\mathbb{D}_{\succ 0} \coloneqq \{a \in \mathbb{D} : a \succ 0\}$, and for every set $X$, let $\mathbb{D}^X$ denote the set of vectors $\mathbb{D}^X \coloneqq \{\boldsymbol{v} \mid \boldsymbol{v} \colon X \to \mathbb{D}\}$.

We naturally extend operations componentwise. In particular, $(\boldsymbol{u} + \boldsymbol{v})(x) := \boldsymbol{u}(x) + \boldsymbol{v}(x)$ for every $x \in X$, and $\boldsymbol{u} \geq \boldsymbol{v}$ iff $\boldsymbol{u}(x) \geq \boldsymbol{v}(x)$ for every $x \in X$.

*Graphs.* A *(labeled directed) graph* is a triple $G = (V, E, A)$, where $V$ is a set of *nodes*, $A$ is a finite set of elements called *actions*, and $E \subseteq V \times A \times V$ is the set of *edges* labeled by actions. We say that $G$ has *finite out-degree* if the set of outgoing edges $\{(w, a, w') \in E : w = v\}$ is finite for every $v \in V$. Similarly, it has *finite in-degree* if the set of ingoing edges is finite for every $v \in V$. If $G$ has both finite out- and in-degree, then we say that $G$ is *locally finite.* A *path* $\pi$ is a finite sequence of nodes $(v_i)_{1 \leq i \leq n}$ and actions $(a_i)_{1 \leq i < n}$ such that $(v_i, a_i, v_{i+1}) \in E$ for all $1 \leq i < n$. We say that $\pi$ is a *path from $v$ to $w$* (or a *$v$-$w$ path*) if $v = v_1$ and $w = v_n$, and its *label* is $a_1 a_2 \cdots a_{n-1}$, where $\varepsilon$ denotes the empty sequence.

A *weighted* graph is a tuple $G = (V, E, A, \mu)$ where $(V, E, A)$ is a graph with a *weight function* $\mu \colon E \to \mathbb{Q}_{>0}$. The *weight* of path $\pi$ is the weight of its edges, *i.e.* $\mu(\pi) := \sum_{1 \leq i < n} \mu(v_i, a_i, v_{i+1})$. A *shortest path from $v$ to $w$* is a $v$-$w$ path $\pi$ minimizing $\mu(\pi)$. We define $\text{dist}_G \colon V \times V \to \mathbb{Q}_{\geq 0} \cup \{\infty\}$ as the *distance function* where $\text{dist}_G(v, w)$ is the weight of a shortest path from $v$ to $w$, with $\text{dist}_G(v, w) := \infty$ if there is none. We assume throughout the paper that weighted graphs have a *minimal weight*, *i.e.* that $\min\{\mu(e) : e \in E\}$ exists. For graphs with finite out-degree, this ensures that if a path exists between two nodes, then a shortest one exists.[4] This mild assumption always holds in our setting.

*Petri nets.* A *weighted Petri net* is a tuple $\mathcal{N} = (P, T, f, \lambda)$ where

- $P$ is a finite set whose elements are called *places*,
- $T$ is a finite set, disjoint from $P$, whose elements are called *transitions*,
- $f \colon (P \times T) \cup (T \times P) \to \mathbb{N}$ is the *flow function* assigning multiplicities to arcs connecting places and transitions, and
- $\lambda \colon T \to \mathbb{Q}_{>0}$ is the *weight function* assigning weights to transitions.

A *marking* is a vector $\boldsymbol{m} \in \mathbb{N}^P$ which indicates that place $p$ holds $\boldsymbol{m}(p)$ *tokens*. A weighted Petri net with $\lambda(t) = 1$ for each $t \in T$ is called a *Petri net.* For example, Figure 1 depicts a Petri net $\mathcal{N}$ with $P = \{p_1, p_2\}$, $T = \{t_1, t_2, t_3\}$, $f(p_1, t_3) = f(p_1, t_2) = f(t_1, p_1) = f(t_2, p_1) = f(t_2, p_2) = 1$ (multiplicity omitted on arcs) and $f(-, -) = 0$ elsewhere (no arc). Moreover, $\mathcal{N}$ is marked with $[p_1 \colon 1, p_2 \colon 0]$.

The *guard* and *effect* of a transition $t \in T$ are vectors $\boldsymbol{g}_t \in \mathbb{N}^P$ and $\boldsymbol{\Delta}_t \in \mathbb{Z}^P$ where $\boldsymbol{g}_t(p) := f(p, t)$ and $\boldsymbol{\Delta}_t(p) := f(t, p) - f(p, t)$. We say that $t$ is *firable* from marking $\boldsymbol{m}$ if $\boldsymbol{m} \geq \boldsymbol{g}_t$. If $t$ is firable from $\boldsymbol{m}$, then it may be *fired*, which leads to marking $\boldsymbol{m}' := \boldsymbol{m} + \boldsymbol{\Delta}_t$. We write this as $\boldsymbol{m} \xrightarrow{t}_{\mathbb{N}} \boldsymbol{m}'$. These notions naturally extend to sequences of transitions, *i.e.* $\xrightarrow{\varepsilon}_{\mathbb{N}}$ denotes the identity relation over $\mathbb{N}^P$, $\boldsymbol{\Delta}_\varepsilon := \boldsymbol{0}$, $\lambda(\varepsilon) := 0$, and for every $t_1, t_2, \ldots, t_k \in T$: $\boldsymbol{\Delta}_{t_1 t_2 \cdots t_k} := \boldsymbol{\Delta}_{t_1} + \boldsymbol{\Delta}_{t_2} + \cdots + \boldsymbol{\Delta}_{t_k}$, $\lambda(t_1 t_2 \cdots t_k) := \lambda(t_1) + \lambda(t_2) + \cdots + \lambda(t_k)$, and

$$\xrightarrow{t_1 t_2 \cdots t_k}_{\mathbb{N}} := \xrightarrow{t_k}_{\mathbb{N}} \circ \cdots \circ \xrightarrow{t_2}_{\mathbb{N}} \circ \xrightarrow{t_1}_{\mathbb{N}} .$$

[4] Otherwise, there could be increasingly better paths, *e.g.* of weights $1, 1/2, 1/4, \ldots$.

We say that $\rightarrow_{\mathbb{N}} := \cup_{t \in T} \xrightarrow{t}_{\mathbb{N}}$ and $\xrightarrow{*}_{\mathbb{N}} := \cup_{\sigma \in T^*} \xrightarrow{\sigma}_{\mathbb{N}}$ are the *step* and *reachability* relations. Note that the latter is the reflexive transitive closure of $\rightarrow_{\mathbb{N}}$.

For example, $\boldsymbol{m} \xrightarrow{t_2 t_3}_{\mathbb{N}} \boldsymbol{m}'$ and $\boldsymbol{m} \xrightarrow{t_1 t_2 t_3 t_3}_{\mathbb{N}} \boldsymbol{m}'$ in Figure 1, where $\boldsymbol{m} := [p_1\colon 1, p_2\colon 0]$ and $\boldsymbol{m}' := [p_1\colon 0, p_2\colon 1]$. Moreover, $t_2$ is not firable in $\boldsymbol{m}'$.

Given a sequence $\sigma \in T^*$, denote by $|\sigma|_t \in \mathbb{N}$ the number of times transition $t$ occurs in $\sigma$. The *Parikh image* of $\sigma$ is the vector $\boldsymbol{\sigma} \in \mathbb{N}^T$ that captures the number of occurrences of transitions appearing in $\sigma$, *i.e.* $\boldsymbol{\sigma}(t) := |\sigma|_t$ for all $t \in T$.

Each weighted Petri net $\mathcal{N} = (P, T, f, \lambda)$ induces a locally finite weighted graph $G_{\mathbb{N}}(\mathcal{N}) := (V, E, T, \mu)$, called its *reachability graph*, where $V := \mathbb{N}^P$, $E := \{(\boldsymbol{m}, t, \boldsymbol{m}') : \boldsymbol{m} \xrightarrow{t}_{\mathbb{N}} \boldsymbol{m}'\}$ and $\mu(\boldsymbol{m}, t, \boldsymbol{m}') := \lambda(t)$ for each $(\boldsymbol{m}, t, \boldsymbol{m}') \in E$. An example of a reachability graph is given on the right of Figure 1. We write $\text{dist}_{\mathcal{N}}$ to denote $\text{dist}_{G_{\mathbb{N}}(\mathcal{N})}$. We have $\text{dist}_{\mathcal{N}}(\boldsymbol{m}, \boldsymbol{m}') \neq \infty$ iff $\boldsymbol{m} \xrightarrow{\sigma}_{\mathbb{N}} \boldsymbol{m}'$ for some $\sigma \in T^*$, and if the latter holds, then $\text{dist}_{\mathcal{N}}(\boldsymbol{m}, \boldsymbol{m}')$ is the minimal weight among such firing sequences $\sigma$. Moreover, for (unweighted) Petri nets, $\text{dist}_{\mathcal{N}}(\boldsymbol{m}, \boldsymbol{m}')$ is the minimal number of transitions to fire to reach $\boldsymbol{m}'$ from $\boldsymbol{m}$.

## 3 Directed Search Algorithms

Our approach relies on classical pathfinding procedures guided by node selection strategies. Their generic scheme is described in Algorithm 1. Its termination with a value $d \neq \infty$ indicates that the weighted graph $G = (V, E, A, \mu)$ has a path from $s$ to $t$ of weight $d$, whereas termination with $d = \infty$ signals that $\text{dist}_G(s, t) = \infty$.

1 $g := [s \mapsto 0, v \mapsto \infty : v \neq s]$
2 $C := \{s\}$
3 **while** $C \neq \emptyset$ **do**
4     $v := \arg\min_{v \in C} S(g, v)$
5     **if** $v = t$ **then return** $g(t)$
6     **for** $(v, a, w) \in E$ **do**
7         **if** $g(v) + \mu(v, a, w) < g(w)$ **then**
8             $g(w) := g(v) + \mu(v, a, w)$
9             $C \quad := C \cup \{w\}$
10     $C := C \setminus \{v\}$
11 **return** $\infty$

**Algorithm 1:** Directed search algorithm.

Algorithm 1 maintains a set of *frontier nodes* $C$ and a mapping $g\colon V \to \mathbb{Q}_{\geq 0} \cup \{\infty\}$ such that $g(w)$ is the weight of the best known path from $s$ to $w$. In Line 4, a *selection strategy* $S$ determines which node $v$ to *expand* next. Starting from Line 6, a successor $w$ of $v$ is added to the frontier if its distance improves.

Let $h\colon V \to \mathbb{Q}_{\geq 0} \cup \{\infty\}$ estimate the distance from all nodes to a target $t \in V$. The selection strategies sending $(g, v)$ respectively to $g(v)$, $g(v) + h(v)$ or $h(v)$ yield the classical Dijkstra's, $\mathsf{A}^*$ and greedy best-first search (*GBFS*) algorithms.

When instantiating $S$ with Dijkstra's selection strategy, a return value $d \neq \infty$ is guaranteed to equal $\text{dist}_G(s, t)$. This is not true for $\mathsf{A}^*$ and GBFS. However, if $h$ fulfills the following *consistency* properties, then $\mathsf{A}^*$ also has this guarantee: $h(t) = 0$ and $h(v) \leq \mu(v, a, w) + h(w)$ for every $(v, a, w) \in E$ (see, *e.g.*, [52]).

In the setting of infinite graphs, unlike GBFS, $\mathsf{A}^*$ and Dijkstra's selection strategies guarantee termination if $\text{dist}_G(s, t) \neq \infty$. Yet, we introduce *unbounded heuristics* for which termination is also guaranteed for GBFS. Note that these guarantees would vanish in the presence of zero weights. An *infinite path* $\pi$ is a sequence of nodes $(v_i)_{i \in \mathbb{N}}$ and actions $(a_i)_{i \in \mathbb{N}}$ such that $(v_i, a_i, v_{i+1}) \in E$ for all $i \in \mathbb{N}$. We say that heuristic $h$ is *unbounded* (w.r.t. $G$) if for every infinite simple path $v_0, v_1, v_2, \ldots$ of $G$ and for every $b \in \mathbb{Q}_{\geq 0}$, there exists an index $i$ s.t. $h(v_i) \geq b$. In other words, unboundedness forbids an infinite simple path of $G$ to "cap" at some distance estimate $b$. The following technical lemma enables to prove termination of GFBS in the presence of unbounded heuristics.

**Lemma 1.** *If $G$ is locally finite, then the following holds:*

1. *The set of paths of weight at most $c \in \mathbb{Q}_{\geq 0}$ starting from node $s$ is finite.*
2. *Let $W \subseteq V$. The set $\text{dist}_G(W, t) := \{\text{dist}_G(w, t) : w \in W\}$ has a minimum.*
3. *No node is expanded infinitely often by Algorithm 1.*

**Theorem 1.** *Algorithm 1 with the greedy best-first search selection strategy always finds reachable targets for locally finite graphs and unbounded heuristics.*

*Proof.* First observe that Algorithm 1 satisfies this invariant:

$$\text{if } g(v) \neq \infty, \text{ then } g(v) \text{ is the weight of a path from } s \text{ to } v \text{ in } G \text{ whose nodes were all expanded, except possibly } v. \quad (*)$$

Assume $\text{dist}_G(s, t) \neq \infty$. For the sake of contradiction, suppose $t$ is never expanded. Let $K_i$ be the subgraph of $G$ induced by nodes expanded at least once within the first $i$ iterations of the **while** loop. In particular, $K_1$ is the graph made only of node $s$. Let $K = K_1 \cup K_2 \cup \cdots$. By Lemma 1 (3), no node is expanded infinitely often, hence $K$ is infinite. Moreover, $K$ has finite out-degree, and each node of $K$ is reachable from $s$ in $K$ by $(*)$. Thus, by König's lemma, $K$ contains an infinite path $v_0, v_1, \ldots \in V$ of pairwise distinct nodes.

Let $w$ be a node of $K$ minimizing $\text{dist}_G(w, t)$. That minimum is well-defined by Lemma 1 (2). Since $s \in K_1 \subseteq K$ and $t$ is reachable from $s$, we have $\text{dist}_G(w, t) \leq \text{dist}_G(s, t) < \infty$. By minimality of $w \neq t$, there exists an edge $(w, a, w')$ of $G$ such that $\text{dist}_G(w', t) < \text{dist}_G(w, t)$ and $w'$ does not appear in $K$. Note that $w'$ is added to $C$ at some point, but is never expanded as it would otherwise belong to $K$. Let $i$ be the smallest index such that $w$ belongs to $K_i$. Since $h$ is unbounded, there exists $j$ such that $h(v_j) > h(w')$ and $v_j$ is expanded after iteration $i$ of the while loop. This is a contradiction as $w'$ would have been expanded instead of $v_j$. □

## 4 Directed Reachability

In this section, we explain how to instantiate Algorithm 1 for finding short(est) firing sequences witnessing reachability in weighted Petri nets. Since Dijkstra's selection strategy does not require any heuristic, we focus on $\mathsf{A}^*$ and greedy best-first search which require consistent and unbounded heuristics. More precisely, we introduce distance under-approximations (Section 4.1); present relevant concrete distance under-approximations (Section 4.2); and put everything together into our framework (Section 4.3).

### 4.1 Distance Under-approximations

A *distance under-approximation* of a weighted Petri net $\mathcal{N} = (P, T, f, \lambda)$ is a function $d \colon \mathbb{N}^P \times \mathbb{N}^P \to \mathbb{Q}_{\geq 0} \cup \{\infty\}$ such that for all $\boldsymbol{m}, \boldsymbol{m}', \boldsymbol{m}'' \in \mathbb{N}^P$:

- $d(\boldsymbol{m}, \boldsymbol{m}') \leq \text{dist}_{\mathcal{N}}(\boldsymbol{m}, \boldsymbol{m}')$,
- $d(\boldsymbol{m}, \boldsymbol{m}'') \leq d(\boldsymbol{m}, \boldsymbol{m}') + d(\boldsymbol{m}', \boldsymbol{m}'')$ (*triangle inequality*), and
- $d$ is effective, *i.e.* there is an algorithm that evaluates $d$ on all inputs.

We naturally obtain a heuristic from $d$ for a directed search towards marking $\boldsymbol{m}_{\text{target}}$. Indeed, let $h \colon \mathbb{N}^P \to \mathbb{Q}_{\geq 0} \cup \{\infty\}$ be defined by $h(\boldsymbol{m}) := d(\boldsymbol{m}, \boldsymbol{m}_{\text{target}})$. The following proposition shows that $h$ is a suitable heuristic for $\mathsf{A}^*$:

**Proposition 1.** *Mapping $h$ is a consistent heuristic.*

*Proof.* Let $\boldsymbol{m}, \boldsymbol{m}' \in \mathbb{N}^P$ and $t \in T$ be such that $\boldsymbol{m} \xrightarrow{t}_{\mathbb{N}} \boldsymbol{m}'$. We have:

$$\begin{aligned}
h(\boldsymbol{m}) &= d(\boldsymbol{m}, \boldsymbol{m}_{\text{target}}) && \text{(by def. of } h\text{)} \\
&\leq d(\boldsymbol{m}, \boldsymbol{m}') + d(\boldsymbol{m}', \boldsymbol{m}_{\text{target}}) && \text{(by the triangle inequality)} \\
&\leq \text{dist}_{\mathcal{N}}(\boldsymbol{m}, \boldsymbol{m}') + d(\boldsymbol{m}', \boldsymbol{m}_{\text{target}}) && \text{(by distance under-approximation)} \\
&\leq \lambda(t) + d(\boldsymbol{m}', \boldsymbol{m}_{\text{target}}) && \text{(since } \boldsymbol{m} \xrightarrow{t}_{\mathbb{N}} \boldsymbol{m}'\text{)} \\
&= \lambda(t) + h(\boldsymbol{m}') && \text{(by def. of } h\text{).}
\end{aligned}$$

Moreover, $h(\boldsymbol{m}_{\text{target}}) = d(\boldsymbol{m}_{\text{target}}, \boldsymbol{m}_{\text{target}}) \leq \text{dist}_{\mathcal{N}}(\boldsymbol{m}_{\text{target}}, \boldsymbol{m}_{\text{target}}) = 0$, where the last equality follows from the fact that weights are positive. ☐

### 4.2 From Petri Net Relaxations to Distance Under-approximations

We now introduce classical relaxations of Petri nets which over-approximate reachability and consequently give rise to distance under-approximations. The main source of hardness of the reachability problem stems from the fact that places are required to hold a non-negative number of tokens. If we relax this requirement and allow negative numbers of tokens, we obtain a more tractable relation. More precisely, we write $\boldsymbol{m} \xrightarrow{t}_{\mathbb{Z}} \boldsymbol{m}'$ iff $\boldsymbol{m}' = \boldsymbol{m} + \boldsymbol{\Delta}_t$. Note that transitions are always firable under this semantics. Moreover, they may lead to "markings" with negative components.

Another source of hardness comes from the fact that markings are discrete. Hence, we can further relax $\to_{\mathbb{Z}}$ into $\to_{\mathbb{Q}}$ where transitions may be scaled down:

$$\boldsymbol{m} \xrightarrow{t}_{\mathbb{Q}} \boldsymbol{m}' \iff \boldsymbol{m}' = \boldsymbol{m} + \delta \cdot \boldsymbol{\Delta}_t \text{ for some } 0 < \delta \leq 1.$$

One gets a less crude relaxation from considering *nonnegative* "markings" only:

$$\boldsymbol{m} \xrightarrow{t}_{\mathbb{Q}_{\geq 0}} \boldsymbol{m}' \iff (\boldsymbol{m} \geq \delta \cdot \boldsymbol{g}_t) \text{ and } (\boldsymbol{m}' = \boldsymbol{m} + \delta \cdot \boldsymbol{\Delta}_t) \text{ for some } 0 < \delta \leq 1.$$

Under these, we obtain "markings" from $\mathbb{Q}^P$ and $\mathbb{Q}^P_{\geq 0}$ respectively. Petri nets equipped with relation $\to_{\mathbb{Q}_{\geq 0}}$ are known as *continuous Petri nets* [14,15].

To unify all three relaxations, we sometimes write $\boldsymbol{m} \xrightarrow{\delta t}_{\mathbb{G}} \boldsymbol{m}'$ to emphasize the scaling factor $\delta$, where $\delta = 1$ whenever $\mathbb{G} = \mathbb{Z}$. Let $d_{\mathbb{G}} \colon \mathbb{N}^P \times \mathbb{N}^P \to \mathbb{Q}_{\geq 0} \cup \{\infty\}$ be defined as $d_{\mathbb{G}}(\boldsymbol{m}, \boldsymbol{m}') \coloneqq \infty$ if $\boldsymbol{m} \overset{*}{\not\to}_{\mathbb{G}} \boldsymbol{m}'$, and otherwise:

$$d_{\mathbb{G}}(\boldsymbol{m}, \boldsymbol{m}') \coloneqq \min \left\{ \sum_{i=1}^{n} \delta_i \cdot \lambda(t_i) : \boldsymbol{m} \xrightarrow{\delta_1 t_1 \cdots \delta_n t_n}_{\mathbb{G}} \boldsymbol{m}' \right\}.$$

In words, $d_{\mathbb{G}}(\boldsymbol{m}, \boldsymbol{m}')$ is the weight of a shortest path from $\boldsymbol{m}$ to $\boldsymbol{m}'$ in the graph induced by the relaxed step relation $\to_{\mathbb{G}}$, where weights are scaled accordingly.

We now show that any $d_{\mathbb{G}}$, which we call the $\mathbb{G}$-*distance*, is a distance under-approximation, and first show effectiveness of all $d_{\mathbb{G}}$. It is well-known and readily seen that reachability over $\mathbb{G} \in \{\mathbb{Z}, \mathbb{Q}\}$ is characterized by the following *state equation*, since transitions are always firable due to the absence of guards:

$$\boldsymbol{m} \xrightarrow{*}_{\mathbb{G}} \boldsymbol{m}' \iff \exists \boldsymbol{\sigma} \in \mathbb{G}^T_{\geq 0} : \boldsymbol{m}' = \boldsymbol{m} + \sum_{t \in T} \boldsymbol{\sigma}(t) \cdot \boldsymbol{\Delta}_t.$$

Here, $\boldsymbol{\sigma}$ can be seen as the Parikh image of a sequence $\sigma$ leading from $\boldsymbol{m}$ to $\boldsymbol{m}'$.

**Proposition 2.** *The functions $d_{\mathbb{Z}}$, $d_{\mathbb{Q}}$, $d_{\mathbb{Q}_{\geq 0}}$ are effective.*

*Proof.* By the state equation, we have:

$$d_{\mathbb{G}}(\boldsymbol{m}, \boldsymbol{m}') = \min \left\{ \sum_{t \in T} \lambda(t) \cdot \boldsymbol{\sigma}(t) : \boldsymbol{\sigma} \in \mathbb{G}^T_{\geq 0}, \boldsymbol{m}' = \boldsymbol{m} + \sum_{t \in T} \boldsymbol{\sigma}(t) \cdot \boldsymbol{\Delta}_t \right\}.$$

Therefore, $d_{\mathbb{Q}}(\boldsymbol{m}, \boldsymbol{m}')$ (resp. $d_{\mathbb{Z}}(\boldsymbol{m}, \boldsymbol{m}')$) are computable by (resp. integer) linear programming, which is complete for P (resp. NP), in its variant where one must check whether the minimal solution is at most some bound.

For $d_{\mathbb{Q}_{\geq 0}}$, note that the reachability relation of a continuous Petri net can be expressed in the existential fragment of linear real arithmetic [8]. Hence, effectiveness follows from the decidability of linear real arithmetic. □

Altogether, we conclude that $d_{\mathbb{G}}$ is a distance under-approximation. Furthermore, we can show that $d_{\mathbb{G}}$ yields *unbounded* heuristics, which, by Theorem 1, ensure termination of GBFS on reachable instances:

**Theorem 2.** *Let $\mathbb{G} \in \{\mathbb{Z}, \mathbb{Q}, \mathbb{Q}_{\geq 0}\}$, then $d_{\mathbb{G}}$ is a distance under-approximation. Moreover, the heuristics arising from it are unbounded.*

*Proof.* Let $\mathcal{N} = (P, T, f, \lambda)$ be a weighted Petri net. Effectiveness of $d_{\mathbb{G}}$ follows from Proposition 2. By definitions and a simple induction, $\xrightarrow{\sigma}_{\mathbb{N}} \subseteq \xrightarrow{\sigma}_{\mathbb{G}}$ for any sequence $\sigma \in T^*$, with weights left unchanged for unscaled transitions. This implies that $d_{\mathbb{G}}(\boldsymbol{m}, \boldsymbol{m}') \leq \mathrm{dist}_{\mathcal{N}}(\boldsymbol{m}, \boldsymbol{m}')$ for every $\boldsymbol{m}, \boldsymbol{m}' \in \mathbb{G}^P$. Moreover, the triangle inequality holds since for every $\boldsymbol{m}, \boldsymbol{m}', \boldsymbol{m}'' \in \mathbb{G}^P$ and sequences $\sigma, \sigma'$:

$$\boldsymbol{m} \xrightarrow{\sigma}_{\mathbb{G}} \boldsymbol{m}' \xrightarrow{\sigma'}_{\mathbb{G}} \boldsymbol{m}'' \text{ implies } \boldsymbol{m} \xrightarrow{\sigma\sigma'}_{\mathbb{G}} \boldsymbol{m}''.$$

Let us sketch the proof of the second part. Let $\boldsymbol{m}_{\text{target}}$ be a marking and let $h_{\mathbb{G}}$ be the heuristic obtained from $d_{\mathbb{G}}$ for $\boldsymbol{m}_{\text{target}}$. Since $h_{\mathbb{Q}}(\boldsymbol{m}) \leq h_{\mathbb{G}}(\boldsymbol{m})$ for all $\boldsymbol{m}$ and $\mathbb{G} \in \{\mathbb{Z}, \mathbb{Q}_{\geq 0}\}$, it suffices to prove that $d_{\mathbb{Q}}$ is unbounded. Suppose it is not. There exist $b \in \mathbb{Q}_{\geq 0}$ and pairwise distinct markings $\boldsymbol{m}_0, \boldsymbol{m}_1, \ldots$ each with $h_{\mathbb{Q}}(\boldsymbol{m}_i) \leq b$. Let $\boldsymbol{x}_i$ be a solution to the state equation that gives $h_{\mathbb{Q}}(\boldsymbol{m}_i)$. By well-quasi-ordering and pairwise distinctness, there is a subsequence such that $\boldsymbol{m}_{i_0}(p) < \boldsymbol{m}_{i_1}(p) < \cdots$ for some $p \in P$. Thus, $\lim_{j \to \infty} \boldsymbol{m}_{\text{target}}(p) - \boldsymbol{m}_{i_j}(p) = -\infty$, and hence $\lim_{j \to \infty} \boldsymbol{x}_{i_j}(s) = \infty$ for some $s \in T$ with $\boldsymbol{\Delta}_s(p) < 0$. This means that $b \geq h_{\mathbb{Q}}(\boldsymbol{m}_{i_j}) = \sum_{t \in T} \lambda(t) \cdot \boldsymbol{x}_{i_j}(t) > b$ for a sufficiently large $j$. □

### 4.3 Directed Reachability Based on Distance Under-approximations

We have all the ingredients to use Algorithm 1 for answering reachability queries.

A *distance under-approximation scheme* is a mapping $\mathcal{D}$ that associates a distance under-approximation $\mathcal{D}(\mathcal{N})$ to each weighted Petri net $\mathcal{N}$. Let $h_{\mathcal{D}(\mathcal{N}),\boldsymbol{m}_{\text{target}}}$ be the heuristic obtained from $\mathcal{D}(\mathcal{N})$ for marking $\boldsymbol{m}_{\text{target}}$. By instantiating Algorithm 1 with this heuristic, we can search for a short(est) firing sequence witnessing that $\boldsymbol{m}_{\text{target}}$ is reachable. Of course, constructing the reachability graph of $\mathcal{N}$ would be at least as difficult as answering this query, or impossible if it is infinite. Hence, we provide $G_{\mathbb{N}}(\mathcal{N})$ *symbolically* through $\mathcal{N}$ and let Algorithm 1 explore it on-the-fly by progressively firing its transitions.

For each $\mathbb{G} \in \{\mathbb{Z}, \mathbb{Q}, \mathbb{Q}_{\geq 0}\}$, the function $\mathcal{D}_{\mathbb{G}}$ mapping a weighted Petri net $\mathcal{N}$ to its $\mathbb{G}$-distance $d_{\mathbb{G}}$ is a distance under-approximation scheme with consistent and unbounded heuristics by Proposition 1, Theorem 1 and Theorem 2. Although Algorithm 1 is geared towards finding paths, it can prove *non*-reachability even for infinite reachability graphs. Indeed, at some point, every candidate marking $\boldsymbol{m} \in C$ may be such that $h_{\mathcal{D}(\mathcal{N}),\boldsymbol{m}_{\text{target}}}(\boldsymbol{m}) = \infty$, which halts with $\infty$. There is no guarantee that this happens, but, as reported *e.g.* by [23,8], the $\mathbb{G}$-distance for domains $\mathbb{G} \in \{\mathbb{Z}, \mathbb{Q}, \mathbb{Q}_{\geq 0}\}$ does well for witnessing non-reachability in practice, often from the very first marking $\boldsymbol{m}_{\text{init}}$.

*An example.* We illustrate our approach with a toy example and $\mathcal{D}_{\mathbb{Q}}$ (the scheme based on the state equation over $\mathbb{Q}^T_{\geq 0}$). Consider the Petri net $\mathcal{N}$ illustrated on the left of Figure 1, but marked with $\boldsymbol{m}_{\text{init}} \coloneqq [p_1\colon 0, p_2\colon 0]$. Suppose we wish to determine whether $\boldsymbol{m}_{\text{init}}$ can reach marking $\boldsymbol{m}_{\text{target}} \coloneqq [p_1\colon 0, p_2\colon 1]$ in $\mathcal{N}$.

We consider the case where Algorithm 1 follows a greedy best-first search, but the markings would be expanded in the same way with $\mathsf{A}^*$. Let us abbreviate a marking $[p_1\colon x, p_2\colon y]$ as $(x, y)$. Since $\boldsymbol{\Delta}_{t_2} = (0, 1)$, the heuristic considers that $\boldsymbol{m}_{\text{init}}$ can reach $\boldsymbol{m}_{\text{target}}$ in a single step using transition $t_2$ (it is unaware of the guard). Marking $(1, 0)$ is expanded and its heuristic value increases to 2 as the state equation considers that both $t_2$ and $t_3$ must be fired (in some unknown order). Markings $(2, 0)$ and $(1, 1)$ are both discovered with respective heuristic values 3 and 1. The latter is more promising, so it is expanded and target $(0, 1)$ is discovered. Since its heuristic value is 0, it is immediately expanded and the correct distance $\text{dist}_{\mathcal{N}}(\boldsymbol{m}_{\text{init}}, \boldsymbol{m}_{\text{target}}) = 3$ is returned. Note that, in this example, the only markings expanded are precisely those occurring on the shortest path.

*Handling multiple targets.* Algorithm 1 can be adapted to search for *some* marking from a given target set $X \subseteq \mathbb{N}^P$. The idea consists simply in using a heuristic $h_X : \mathbb{N}^P \to \mathbb{Q}_{\geq 0} \cup \{\infty\}$ estimating the weight of a shortest path to *any* target:

$$h_X(\boldsymbol{m}) \coloneqq \min\{h_{\mathcal{D}(\mathcal{N}),\boldsymbol{m}_{\text{target}}}(\boldsymbol{m}) : \boldsymbol{m}_{\text{target}} \in X\}.$$

This is convenient for partial reachability instances occurring in practice, *i.e.*

$$X \coloneqq \left\{\boldsymbol{m}_{\text{target}} \in \mathbb{N}^P : \boldsymbol{m}_{\text{target}}(p) \sim_p \boldsymbol{c}(p)\right\} \text{ where } \boldsymbol{c} \in \mathbb{N}^P \text{ and } \textit{each} \sim_p \in \{=, \geq\}.$$

## 5 Experimental Results

We implemented Algorithm 1 in a prototype called FastForward [10], which supports all presented selection strategies and distance under-approximations. We evaluate FastForward empirically with three main goals in mind. First, we show that our approach is competitive with established tools and can even vastly outperform them, and we also give insights on its performance w.r.t. its parameterizations. Second, we compare the length of the witnesses reported by the different tools. Third, we briefly discuss the quality of the heuristics.

**Technical details.** Our tool is written in C# and uses Gurobi [32], a state-of-the-art MILP solver, for distance under-approximations. Benchmarks were run on an machine with an 8-Core Intel® Core™ i7-7700 CPU @ 3.60GHz running Ubuntu 18.04 and with memory constrained to ∼8GB. We used a timeout of 60 seconds per instance, and all tools were invoked from a Python script using the `time` module for time measurements.

A minor challenge arises from the fact that many instances specify an upward-closed set of initial markings rather than a single one. For example, $\boldsymbol{m}_{\text{init}}(p) \geq 1$ to specify, *e.g.*, an arbitrary number of threads. We handle this by setting $\boldsymbol{m}_{\text{init}}(p) = 1$ and adding a transition $t_p$ producing a token into $p$.

As a preprocessing step, we implemented *sign analysis* [29]. It is a general pruning technique running in polynomial time that has been shown beneficial for reducing the size of the state-space of Petri nets. Initially, places that carry tokens are viewed as marked. For each transition whose input places are marked, the output places also become marked. When a fixpoint is reached, places left unmarked cannot carry tokens in any reachable marking, so they are discarded.

**Benchmarks.** Due to the lack of tools handling reachability for *unbounded* state spaces, benchmarks arising in the literature are primarily *coverability* instances[5], *i.e.* reachability towards an upward closed set of target markings. We gathered 61 positive and 115 negative coverability instances originating from five suites [39,28,6,35,18] previously used for benchmarking [23,8,29]. They arise from the analysis of multi-threaded C programs with shared-memory; mutual

[5] The Model Checking Contest focuses on reachability for *finite* state spaces.

exclusion algorithms; communication protocols; provenance analysis in the context of a medical messaging and a bug-tracking system; and the verification of Erlang concurrent programs. We further extracted the *sypet* suite made of 30 positive (standard) reachability instances arising from queries encountered in type-directed program synthesis [24]. The overall goal of this work is to enable a vast range of untapped applications requiring reachability over unbounded state-spaces, rather than just coverability. To obtain further (positive) instances of the Petri net reachability problem, we performed random walks on the Petri nets from the aforementioned coverability benchmarks. To this end, we used the largest quarter of distinct Petri nets from each coverability suite, for a total of 33. We performed one random walk each of lengths 20, 25, 30, 35, 40, 50, 60, 75, 90 and 100, and we saved the resulting marking as the target. For nets with an upward-closed initial marking, we randomly chose to start with a number of tokens between 1 and 20% of the length of the walk. It is important to note that even with long random walks, instances can (and in fact tend to) have short witnesses. To remove trivial instances and only keep the most challenging ones, we removed those instances where any considered tool reported a witness of length at most 20, disregarding the transitions used to generate the initial marking. This leaves us with 127 challenging instances on which the shortest witness is either unknown or has length more than 20. Moreover, this yields real-world Petri nets with no bias towards any specific kind of targets.

This table summarizes the characteristics of the various benchmarks:

| **Suite** | **Size** | **Number of places** | | | | **Number of transitions** | | | |
|---|---|---|---|---|---|---|---|---|---|
| | | min. | med. | mean | max. | min. | med. | mean | max. |
| COVERABILITY | 61 | 16 | 87 | 226 | 2826 | 14 | 181 | 1519 | 27370 |
| SYPET | 30 | 65 | 251 | 320 | 1199 | 537 | 2307 | 2646 | 8340 |
| RANDOM WALKS | 127 | 52 | 306 | 531 | 2826 | 60 | 3137 | 5885 | 27370 |

**Tool comparison.** To evaluate our approach on reachability instances, we compare FastForward to LoLA [53], a tool developed for two decades that wins several categories of the Model Checking Contest every year. LoLA is geared towards model checking of finite state spaces, but it implements semi-decision procedures for the unbounded case. We further compare the three selection strategies of Algorithm 1: $\mathsf{A}^*$, GBFS and Dijkstra; the two first with the distance under-approximation scheme $\mathcal{D}_\mathbb{Q}$, which provides the best trade-off between estimate quality and efficiency. In fact, the other heuristics perform strictly worse on almost all instances. We also considered comparing with KReach [17], a tool showcased at TACAS'20 that implements an exact non-elementary algorithm. However, it timed out on all instances with a larger time limit of 10 minutes.

Figure 2 depicts the number of reachability instances decided by the tools within the time limit. As shown, all approaches outperform LoLA, with GBFS as the clear winner on the RANDOM-WALK suite and $\mathsf{A}^*$ slightly better on the SYPET suite. Note that Dijkstra's selection strategy sometimes competes due

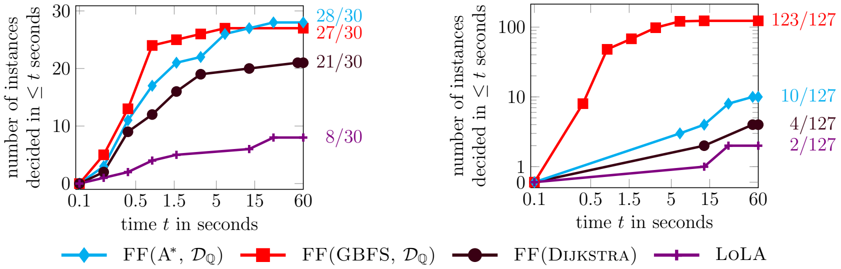

**Fig. 2.** Cumulative number of reachability instances decided over time. *Left*: SYPET suite (semi-log scale). *Right*: RANDOM-WALK suite (log scale).

to its locally very cheap computational cost (no heuristic evaluation), but its performance generally decreases as the distance increases.

To show the versatility of our approach, we also benchmarked FastForward on the original coverability instances. Recall that coverability EXPSPACE-complete and reduces to reachability in linear time [45,51]. While exceeding the PSPACE-completeness of reachability for finite state-spaces [38,21], coverability is much more tame than the non-elementary complexity of (unbounded) reachability. We compare FastForward to four tools implementing algorithms tailored, some of which are specifically to the coverability problem: LoLA, Bfc [39], ICover [29] and the backward algorithm (based on [1]) of MIST [28]. We did not test Petrinizer [23] since it only handles negative instances, while we focus on positive ones; likewise for QCover [8] since it is superseded by ICover.

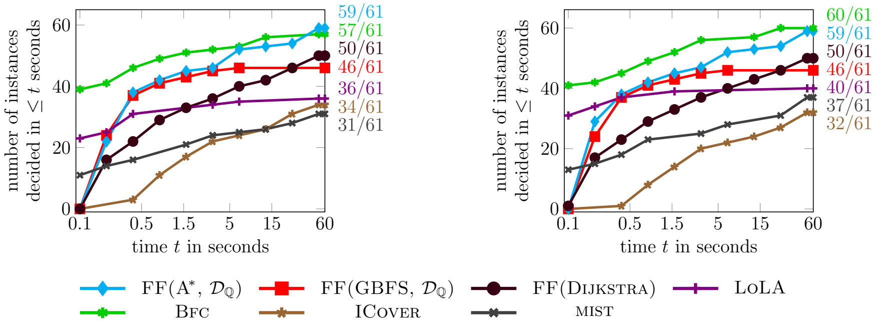

**Fig. 3.** Cumulative number of (positive) coverability instances decided over time. *Left*: Evaluation on the original instances. *Right*: Evaluation on the pre-pruned instances.

Figure 3 illustrates the number of coverability instances decided within the time limit. The left side corresponds to an evaluation on the original instances where FastForward performs pruning (included in its runtime). On the righthand side the pruned instances are the input for all tools, and the time for this pruning is not included for any tool. As a caveat, ICover performs its own preprocessing which includes pruning among techniques specific to coverability. This preprocessing is enabled (and its time is included) even when pruning is already done. Using FastForward(A$^*$, $\mathcal{D}_\mathbb{Q}$), we decide more instances than all tools on unpruned Petri nets, and one less than Bfc for pre-pruned instances. It is worth mentioning that with a time limit of 10 minutes per instance, FastForward(A$^*$, $\mathcal{D}_\mathbb{Q}$) is the only tool to decide all 61 instances.

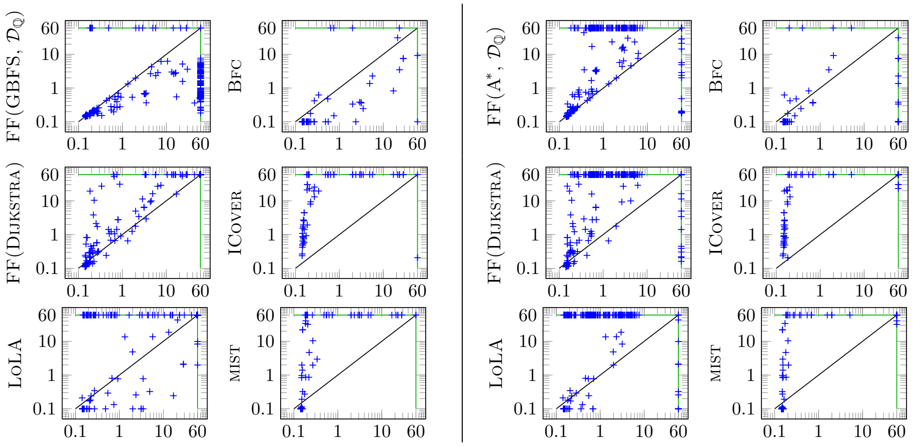

**Fig. 4.** Runtime comparison against FF(A$^*$, $\mathcal{D}_\mathbb{Q}$) (*left*) and FF(GBFS, $\mathcal{D}_\mathbb{Q}$) (*right*), in seconds, for individual instances without pre-pruning. Tools on the first column of each side include coverability and reachability instances, while those on the second column of each side include coverability only. Marks on the green lines denote timeouts (60 s).

We also compared the running time of A$^*$ and GBFS with $\mathcal{D}_\mathbb{Q}$ to the other tools and approaches. For each tool, we considered the type of instances it can handle: either reachability and coverability, or coverability only. Figure 4 depicts this comparison, where the base approach is faster for data points that lie in the upper-left half of the graph. The axes start at 0.1 second to avoid a comparison based on technical aspects such as the programming language. Yet, LoLA, Bfc and mist regularly solve instances faster than this, which speaks to their level of optimization. We can see that FastForward outperforms ICover, LoLA and mist overall. We cannot compete with Bfc in execution time as it is a highly optimized tool specifically tailored to only the coverability problem that can employ optimization techniques such as Karp-Miller trees that do not work for reachability queries.

**Length of the witnesses.** Since our approach is also geared towards the identification of short(est) reachability witnesses, we compared the different tools with respect to length of the reported one, depicted in Figure 5. Positive values on the $y$-axis mean the witness was not minimal, while $y = 0$ means it was. Note that the points for BFC must be taken with a grain of salt: it uses a different file format, and its translation utility can introduce additional transitions. This means that even if BFC found a shortest witness, it could be longer than a shortest one of the original instance.

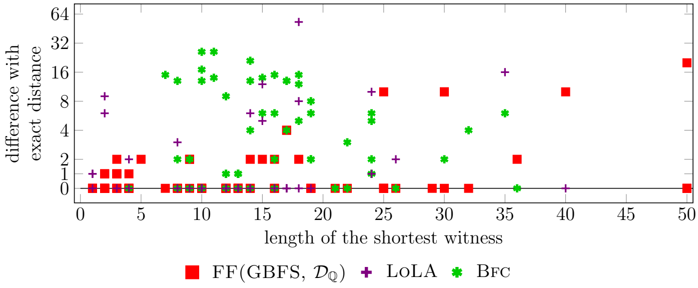

**Fig. 5.** Length of the returned witness, per tool, compared to the length of a shortest witness. ICOVER is left out as it does not return witnesses. FF($A^*$, $\mathcal{D}_{\mathbb{Q}}$), FF(DIJKSTRA) and MIST are left out as they are guaranteed to return shortest witnesses.

Still, the graph shows that reported witnesses can be far from minimal. For example, on one instance LoLA returns a witness that is 53 transitions longer than the one of FASTFORWARD($A^*$, $\mathcal{D}_{\mathbb{Q}}$). Still, LoLA returns a shortest witness on 28 out of 43 instances. Similarly, FASTFORWARD(GBFS, $\mathcal{D}_{\mathbb{Q}}$) finds a shortest path on 60 out of 83 instances[6]. In contrast, MIST finds a shortest witness on all instances since its backward algorithm is guaranteed to do so on unweighted Petri nets, which constitute all of our instances. Again, this approach is tailored to coverability and cannot be lifted to reachability.

**Heuristics and pruning.** We briefly discuss the quality of the heuristics and the impact of pruning. The left-hand side of Figure 6 compares the exact distance to the estimated distance from the initial marking.[7] It shows that it is incredibly accurate for all $\mathbb{G}$-distances, but even more so for $\mathbb{G} = \mathbb{Q}_{\geq 0}$. We experimented with this distance using the logical translation of [8] and Z3 [49] as the optimization modulo theories solver. At present, it appears that the gain in estimate quality does not compensate for the extra computational cost.

As depicted on the right-hand side of Figure 6, pruning can make some instances trivial, but in general, many challenging instances remain so. On average, around 50% of places and 40% of transitions were pruned.

[6] These numbers disregard instances where the tool did not finish or where a shortest witness is not known, *i.e.* no method guaranteeing one finished in time.

[7] Z3 reported two non optimal solutions which explains the two points above the line.

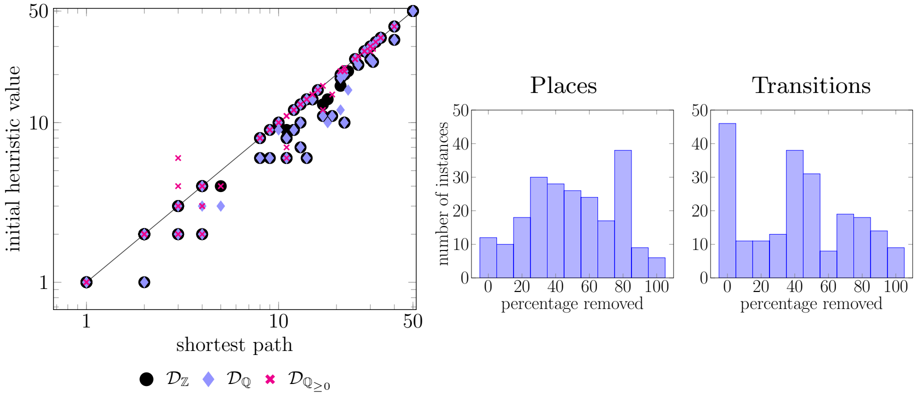

**Fig. 6.** *Left*: initial distance estimation compared to the exact distance (points closer to the diagonal are better). *Right*: number of instances per percentage of places (left) and transitions (right) removed by pruning (rounded to nearest multiple of 10).

## 6 Conclusion

We presented an efficient approach to the Petri net reachability problem that uses state-space over-approximations as distance oracles in the classical graph traversal algorithms $\mathsf{A}^*$ and greedy best-first search. Our experiments have shown that using the state equation over $\mathbb{Q}_{\geq 0}^T$ provides the best trade-off between computational feasibility and the accuracy of the oracle. However, we expect that further advances in optimization modulo theories solvers may enable employing stronger over-approximations such as continuous Petri nets in the future.

Moreover, non-algebraic distance under-approximations also fit naturally in our framework, *e.g.* the syntactic distance of [55] and "$\alpha$-graphs" of [24]. These are crude approximations with low computational cost. Our preliminary tests show that, although they could not compete with our distances, they can provide early speed-ups on instances with large branching factors. An interesting line of research consists in identifying cheap approximations with better estimates.

We wish to emphasize that our approach to the reachability problem has the potential to also be naturally used for semi-deciding reachability in extensions of Petri nets with a recursively enumerable reachability problem, such as Petri nets with resets and transfers [3,19] as well as colored Petri nets [37]. These extensions have, for instance, been used for the generation of program loop invariants [54], the validation of business processes [59] and the verification of multi-threaded C and Java program skeletons with communication primitives [16,39]. Linear rational and integer arithmetic over-approximations for such extended Petri nets exist [12,9,34,31] and could smoothly be used inside our framework.

## Acknowledgments

We thank Juliette Fournis d'Albiat for her help with extracting the SYPET suite.

## References

[1] Abdulla, P.A., Cerans, K., Jonsson, B., Tsay, Y.: General decidability theorems for infinite-state systems. In: Proc. $$\text{11}^\text{th }$$ Annual IEEE Symposium on Logic in Computer Science (LICS). pp. 313–321. IEEE Computer Society (1996). 10.1109/LICS.1996.561359

[2] Angeli, D., De Leenheer, P., Sontag, E.D.: A Petri net approach to the study of persistence in chemical reaction networks. Mathematical Biosciences **210**(2), 598–618 (2007). 10.1016/j.mbs.2007.07.003 10.1016/j.mbs.2007.07.003 17869313

[3] Araki, T., Kasami, T.: Some decision problems related to the reachability problem for Petri nets. Theoretical Computer Science **3**(1), 85–104 (1976). 10.1016/0304-3975(76)90067-0

[4] Athanasiou, K., Liu, P., Wahl, T.: Unbounded-thread program verification using thread-state equations. In: Proc. $${{8}^{\rm th}}$$ International Joint Conference on Automated Reasoning (IJCAR). pp. 516–531. Springer (2016). 10.1007/978-3-319-40229-1_35

[5] Bansal, K., Koskinen, E., Wies, T., Zufferey, D.: Structural counter abstraction. In: Proc. $${{19}^{{\rm th}}}$$ International Conference on Tools and Algorithms for the Construction and Analysis of Systems (TACAS). pp. 62–77. Springer (2013). 10.1007/978-3-642-36742-7_5

[6] Barth, A., Mitchell, J.C., Datta, A., Sundaram, S.: Privacy and utility in business processes. In: Proc. $${{20}^{\rm th}}$$ IEEE Computer Security Foundations Symposium (CSF). pp. 279–294. IEEE Computer Society (2007). 10.1109/CSF.2007.26

[7] Bjørner, N., Phan, A., Fleckenstein, L.: $$\nu $$Z - an optimizing SMT solver. In: Proc. $${{21}^{\rm th}}$$ International Conference on Tools and Algorithms for the Construction and Analysis of Systems (TACAS). pp. 194–199. Springer (2015). 10.1007/978-3-662-46681-0_14

[8] Blondin, M., Finkel, A., Haase, C., Haddad, S.: The logical view on continuous Petri nets. ACM Transactions on Computational Logic (TOCL) **18**(3), 24:1–24:28 (2017). 10.1145/3105908

[9] Blondin, M., Haase, C., Mazowiecki, F.: Affine extensions of integer vector addition systems with states. In: Proc. $${{29}^{\rm th}}$$ International Conference on Concurrency Theory (CONCUR). pp. 14:1–14:17. Schloss Dagstuhl - Leibniz-Zentrum für Informatik (2018). 10.4230/LIPIcs.CONCUR.2018.14

[10] Blondin, M., Haase, C., Offtermatt, P.: Fastforward: A tool for reachability in Petri nets with infinite state spaces. Artifact for the TACAS21 contribution “Directed Reachability for Infinite-State Systems” (2021). 10.6084/m9.figshare.13573592

[11] Chaouiya, C.: Petri net modelling of biological networks. Briefings in Bioinformatics **8**(4), 210–219 (2007). 10.1093/bib/bbm029 10.1093/bib/bbm029 17626066

[12] Chistikov, D., Haase, C., Halfon, S.: Context-free commutative grammars with integer counters and resets. Theoretical Computer Science **735**, 147–161 (2018). 10.1016/j.tcs.2016.06.017

[13] Czerwiński, W., Lasota, S., Lazić, R., Leroux, J., Mazowiecki, F.: The reachability problem for Petri nets is not elementary. In: Proc. $${{51}^{\rm st}}$$ Annual ACM SIGACT Symposium on Theory of Computing (STOC). pp. 24–33. ACM (2019). 10.1145/3313276.3316369

[14] David, R., Alla, H.: Continuous Petri nets. In: Proc. $${{8}^{\rm th}}$$ European Workshop on Application and Theory of Petri nets. vol. 340, pp. 275–294 (1987)

[15] David, R., Alla, H.: Discrete, Continuous, and Hybrid Petri nets. Springer, $${{2}^{\rm nd}}$$ edn. (2010)

[16] Delzanno, G., Raskin, J., Van Begin, L.: Towards the automated verification of multithreaded Java programs. In: Proc. $${{8}^{\rm th}}$$ International Conference on Tools and Algorithms for the Construction and Analysis of Systems (TACAS). pp. 173–187. Springer (2002). 10.1007/3-540-46002-0_13

[17] Dixon, A., Lazić, R.: Kreach: A tool for reachability in Petri nets. In: Proc. $${{26}^{\rm th}}$$ International Conference on Tools and Algorithms for the Construction and Analysis of Systems (TACAS). pp. 405–412. Springer (2020). 10.1007/978-3-030-45190-5_22

[18] D’Osualdo, E., Kochems, J., Ong, C.L.: Automatic verification of erlang-style concurrency. In: Proc. $${{20}^{\rm th}}$$ International Symposium on Static Analysis (SAS). pp. 454–476. Springer (2013). 10.1007/978-3-642-38856-9_24

[19] Dufourd, C., Finkel, A., Schnoebelen, P.: Reset nets between decidability and undecidability. In: Proc. $${{25}^{\rm th}}$$ International Colloquium on Automata, Languages and Programming (ICALP). pp. 103–115. Springer (1998). 10.1007/BFb0055044

[20] Edelkamp, S., Schuppan, V., Bosnacki, D., Wijs, A., Fehnker, A., Aljazzar, H.: Survey on directed model checking. In: Proc. $${{5}^{\rm th}}$$ International Workshop on Model Checking and Artificial Intelligence (MoChArt). pp. 65–89. Springer (2008). 10.1007/978-3-642-00431-5_5

[21] Esparza, J.: Decidability and complexity of Petri net problems — An introduction, pp. 374–428. Springer (1998). 10.1007/3-540-65306-6_20

[22] Esparza, J., Ganty, P., Leroux, J., Majumdar, R.: Verification of population protocols. Acta Informatica **54**(2), 191–215 (2017). 10.1007/s00236-016-0272-3

[23] Esparza, J., Ledesma-Garza, R., Majumdar, R., Meyer, P.J., Nikšić, F.: An SMT-based approach to coverability analysis. In: Proc. $${{26}^{\rm th}}$$ International Conference on Computer Aided Verification (CAV). pp. 603–619. Springer (2014). 10.1007/978-3-319-08867-9_40

[24] Feng, Y., Martins, R., Wang, Y., Dillig, I., Reps, T.W.: Component-based synthesis for complex APIs. In: Proc. $${{44}^{\rm th}}$$ ACM SIGPLAN Symposium on Principles of Programming Languages (POPL). pp. 599–612. ACM (2017). 10.1145/3009837.3009851

[25] Fraca, E., Haddad, S.: Complexity analysis of continuous Petri nets. Fundamenta Informaticae **137**(1), 1–28 (2015). 10.3233/FI-2015-1168

[26] Galenson, J.: Dynamic and Interactive Synthesis of Code Snippets. Ph.D. thesis, University of California (2014)

[27] Galenson, J., Reames, P., Bodík, R., Hartmann, B., Sen, K.: Codehint: dynamic and interactive synthesis of code snippets. In: Proc. $${{36}^{\rm th}}$$ International Conference on Software Engineering (ICSE). pp. 653–663. ACM (2014). 10.1145/2568225.2568250

[28] Ganty, P.: Algorithmes et structures de données efficaces pour la manipulation de contraintes sur les intervalles. Master’s thesis, Université Libre de Bruxelles (2002), (In French)

[29] Geffroy, T., Leroux, J., Sutre, G.: Occam’s razor applied to the Petri net coverability problem. Theoretical Computer Science **750**, 38–52 (2018). 10.1016/j.tcs.2018.04.014

[30] German, S.M., Sistla, A.P.: Reasoning about systems with many processes. Journal of the ACM **39**(3), 675–735 (1992). 10.1145/146637.146681

[31] Gupta, U., Shah, P., Akshay, S., Hofman, P.: Continuous reachability for unordered data Petri nets is in PTime. In: Proc. $${{22}^{\rm nd}}$$ International Conference on Foundations of Software Science and Computation Structures (FoSSaCS). pp. 260–276. Springer (2019). 10.1007/978-3-030-17127-8_15

[32] Gurobi Optimization, L.: Gurobi optimizer reference manual (2020), http://www.gurobi.com

[33] Heiner, M., Gilbert, D.R., Donaldson, R.: Petri nets for systems and synthetic biology. In: Formal Methods for Computational Systems Biology. pp. 215–264. Springer (2008). 10.1007/978-3-540-68894-5_7

[34] Hofman, P., Leroux, J., Totzke, P.: Linear combinations of unordered data vectors. In: Proc. $${{32}^{\rm nd}}$$ Annual ACM/IEEE Symposium on Logic in Computer Science (LICS). pp. 1–11. IEEE Computer Society (2017). 10.1109/LICS.2017.8005065

[35] Janák, J.: Issue Tracking Systems. Master’s thesis, Masaryk University (2009)

[36] Jeng, M.D., Chen, S.C.: A heuristic search approach using approximate solutions to Petri net state equations for scheduling flexible manufacturing systems. International Journal of Flexible Manufacturing Systems **10**(2), 139–162 (1998). 10.1023/A:1008097430956

[37] Jensen, K.: Coloured Petri nets: basic concepts, analysis methods and practical use, vol. 1. Springer Science & Business Media (2013)

[38] Jones, N.D., Landweber, L.H., Lien, Y.E.: Complexity of some problems in Petri nets. Theoretical Computer Science **4**(3), 277–299 (1977). 10.1016/0304-3975(77)90014-7

[39] Kaiser, A., Kroening, D., Wahl, T.: A widening approach to multithreaded program verification. ACM Transactions on Programming Languages and Systems (TOPLAS) **36**(4), 14:1–14:29 (2014). 10.1145/2629608

[40] Kosaraju, S.R.: Decidability of reachability in vector addition systems (preliminary version). In: Proc. $${{14}^{\rm th}}$$ Symposium on Theory of Computing (STOC). pp. 267–281. ACM (1982). 10.1145/800070.802201

[41] Lambert, J.: A structure to decide reachability in Petri nets. Theoretical Computer Science **99**(1), 79–104 (1992). 10.1016/0304-3975(92)90173-D

[42] Lee, D.Y., DiCesare, F.: Scheduling flexible manufacturing systems using Petri nets and heuristic search. IEEE Transactions on robotics and automation **10**(2), 123–132 (1994). 10.1109/70.282537

[43] Leroux, J.: Vector addition systems reachability problem (A simpler solution). In: Turing-100 - The Alan Turing Centenary. pp. 214–228. EasyChair (2012).

[44] Leroux, J., Schmitz, S.: Demystifying reachability in vector addition systems. In: Proc. $${{30}^{\rm th}}$$ Annual ACM/IEEE Symposium on Logic in Computer Science (LICS). pp. 56–67. IEEE Computer Society (2015). 10.1109/LICS.2015.16

[45] Lipton, R.J.: The reachability problem requires exponential space. Tech. rep., Yale University (1976)

[46] Liu, B., Dong, W., Zhang, Y.: Accelerating API-based program synthesis via API usage pattern mining. IEEE Access **7**, 159162–159176 (2019). 10.1109/ACCESS.2019.2950232

[47] Mayr, E.W.: An algorithm for the general Petri net reachability problem. In: Proc. $${{13}^{\rm th}}$$ Symposium on Theory of Computing (STOC). pp. 238–246. ACM (1981). 10.1145/800076.802477

[48] Mejía, G., Odrey, N.G.: An approach using Petri nets and improved heuristic search for manufacturing system scheduling. Journal of Manufacturing Systems **24**(2), 79–92 (2005). 10.1016/S0278-6125(05)80009-3

[49] de Moura, L.M., Bjørner, N.: Z3: an efficient SMT solver. In: Proc. $${{14}^{\rm th}}$$ International Conference on Tools and Algorithms for the Construction and Analysis of Systems (TACAS). pp. 337–340. Springer (2008). 10.1007/978-3-540-78800-3_24, tool available at https://github.com/Z3Prover/z3.

[50] Murata, T.: Petri nets: Properties, analysis and applications. Proceedings of the IEEE **77**(4), 541–580 (1989). 10.1109/5.24143

[51] Rackoff, C.: The covering and boundedness problems for vector addition systems. Theoretical Computer Science **6**, 223–231 (1978). 10.1016/0304-3975(78)90036-1

[52] Russell, S., Norvig, P.: Artificial Intelligence: A Modern Approach. Prentice Hall Press, $${{3}^{\rm rd}}$$ edn. (2009)

[53] Schmidt, K.: LoLA: A low level analyser. In: Proc. International Conference on Application and Theory of Petri Nets (ICATPN). pp. 465–474. Springer (2000). 10.1007/3-540-44988-4_27

[54] Silverman, J., Kincaid, Z.: Loop summarization with rational vector addition systems. In: Proc. $${{31}^{\rm st}}$$ International Conference on Computer Aided Verification (CAV). pp. 97–115. Springer (2019). 10.1007/978-3-030-25543-5_7

[55] Strazny, T.: An algorithmic framework for checking coverability in well-structured transition systems. Ph.D. thesis, Universität Oldenburg (2014), http://csd.informatik.uni-oldenburg.de/~skript/pub/diss/strazny-phdthesis-roterbericht.pdf

[56] Uma, G., Prasad, B.: Reachability trees for Petri nets: a heuristic approach. Knowledge-Based Systems **6**(3), 174 – 177 (1993). 10.1016/0950-7051(93)90042-R

[57] van der Aalst, W.: The application of Petri nets to workflow management. Journal of Circuits, Systems, and Computers **8**(1), 21–66 (1998). 10.1142/S0218126698000043

[58] Watel, D., Weisser, M., Barth, D.: Parameterized complexity and approximability of coverability problems in weighted Petri nets. In: Proc. $${{38}^{\rm th}}$$ International Conference on Application and Theory of Petri Nets and Concurrency (PETRI NETS). pp. 330–349. Springer (2017). 10.1007/978-3-319-57861-3_19

[59] Wynn, M.T., van der Aalst, W.M.P., ter Hofstede, A.H.M., Edmond, D.: Synchronization and cancelation in workflows based on reset nets. International Journal of Cooperative Information Systems **18**(1), 63–114 (2009). 10.1142/S0218843009002002

[60] Yang, C.H., Dill, D.L.: Validation with guided search of the state space. In: Proc. $${{35}^{\rm th}}$$ Conference on Design Automation (DAC). pp. 599–604. ACM (1998). 10.1145/277044.277201

[61] Zuck, L.D., Pnueli, A.: Model checking and abstraction to the aid of parameterized systems (a survey). Computer Languages, Systems & Structures **30**(3-4), 139–169 (2004). 10.1016/j.cl.2004.02.006

